# Co-production of pigment and high value-added bacterial nanocellulose from *Suaeda salsa* biomass with improved efficiency of enzymatic saccharification and fermentation

**DOI:** 10.3389/fbioe.2023.1307674

**Published:** 2023-11-30

**Authors:** Ran Tan, Qiwei Sun, Yiran Yan, Tao Chen, Yifei Wang, Jiakun Li, Xiaohong Guo, Zuoqing Fan, Yao Zhang, Linxu Chen, Guochao Wu, Nan Wu

**Affiliations:** ^1^ Shandong Key Laboratory of Edible Mushroom Technology, School of Agriculture, Ludong University, Yantai, China; ^2^ School of Resources and Environmental Engineering, Ludong University, Yantai, China; ^3^ Shandong Institute of Sericulture, Yantai, China; ^4^ School of Chemistry and Materials Science, Ludong University, Yantai, China; ^5^ State Key Laboratory of Microbial Technology, Shandong University, Qingdao, China; ^6^ Key Laboratory of Molecular Module-Based Breeding of High Yield and Abiotic Resistant Plants in Universities of Shandong, School of Agriculture, Ludong University, Yantai, China

**Keywords:** *Suaeda salsa*, pigment extraction, pretreatment, enzymatic saccharification, bacterial nanocellulose, crystallinity

## Abstract

This study evaluated the co-production of pigment and bacterial nanocellulose (BNC) from *S. salsa* biomass. The extraction of the beet red pigment reduced the salts and flavonoids contents by 82.7%–100%, promoting the efficiencies of enzymatic saccharification of the biomass and the fermentation of BNC from the hydrolysate. SEM analysis revealed that the extraction process disrupted the lignocellulosic fiber structure, and the chemical analysis revealed the lessened cellulase inhibitors, consequently facilitating enzymatic saccharification for 10.4 times. BNC producing strains were found to be hyper-sensitive to NaCl stress, produced up to 400.4% more BNC from the hydrolysate after the extraction. The fermentation results of BNC indicated that the LDU-A strain yielded 2.116 g/L and 0.539 g/L in ES-M and NES-M, respectively. In comparison to the control, the yield in ES-M increased by approximately 20.0%, while the enhancement in NES-M was more significant, reaching 292.6%. After conducting a comprehensive characterization of BNC derived from *S. salsa* through Scanning Electron Microscopy (SEM), Fourier Transform Infrared Spectroscopy (FTIR), X-ray Diffraction (XRD), and Thermogravimetric Analysis (TGA), the average fiber diameter distribution of these four BNC materials ranges from 22.23 to 33.03 nanometers, with a crystallinity range of 77%–90%. Additionally, they exhibit a consistent trend during the thermal degradation process, further emphasizing their stability in high-temperature environments and similar thermal properties. Our study found an efficient co-production approach of pigment and BNC from *S. salsa* biomass. Pigment extraction made biomass more physically and chemically digestible to cellulase, and significantly improved BNC productivity and quality.

## 1 Introduction

*S. salsa* is an annual leafy succulent salt plant with a wide distribution, capable of forming mono-optimal communities on beaches and lakesides. It is mainly distributed in coastal wetlands in the United States, Asia, Latin America, and the Middle East (Cai et al., 2021; Xu et al., 2023), and has a large volume of production, with an estimated annual yield between 900,000 and 1.1 million tons (Ma et al., 2020).

*S. salsa* is a rich source of beet red pigments, a valuable natural pigment used for food additives. Beet red pigments are water-soluble nitrogen-containing pigments (Esatbeyoglu et al., 2015). Beet red pigment was widely used in food industry, healthcare, medicine, cosmetics and other industries (Fu et al., 2020). The residual biomass of *S. salsa*, obtained after the extraction of red pigment, is currently considered as waste, for which no reported utilization has been found in the literature.

BNC is a novel material composed of cellulose nanofibers, forming a complex network that is primarily produced through the fermentation of acetic acid bacteria. BNC exhibits several remarkable features, such as ultrafine fiber diameter, high polymerization degree, high crystallinity, superior water-holding capacity, and excellent biocompatibility (Suárez-Avendaño et al., 2022; Liang, 2023), and is of great of industrial interest (Lee et al., 2014). Moreover, BNC is the finest natural nanomaterial known to date, surpassing plant cellulose (Lee et al., 2021), and is free of the need for laborious purification procedures to eliminate impurities. BNC has a broad range of applications, including papermaking, food packaging, textile industry, bioplastics, wound dressings, and scaffolds for tissue engineering (Vatansever et al., 2019; Gao et al., 2020; Heise et al., 2021; Kedzior et al., 2021).

At present, the production of BNC is constrained by the high cost of raw materials, hindering its widespread application. In response to this challenge, researchers have initiated investigations into alternative, low-cost feedstocks like food waste, agricultural residue, fiber sludge waste, and waste cotton textiles with the aim of economically viable BNC production (Cheng et al., 2017; Dubey et al., 2018; Jahan et al., 2018; Kumar et al., 2019; Ye et al., 2019). However, the cost of the pretreatment and enzymatic saccharification of biomass is normally high, and the hydrolysate in this study contained a large amount of fermentation inhibitors, which reduce the efficiency of enzymatic conversion. Furthermore, economic constraints in the pretreatment and enzymatic saccharification processes of biomass present limitations on its development. Halophyte *S. salsa* could be a potential low-cost feedstock, but it contained a large amount of non-cellulosic substances, including salts, phenolic compounds, and organic acids, that might interfere with or inhibit the enzymatic saccharification or BNC fermentation (Jing et al., 2009; Tejirian and Xu, 2010; Zhai et al., 2018). The halophyte *S. salsa* exhibits promising potential for pigment extraction. In contrast to its status of being discarded, further investigation into its pigment extraction applications reveals a novel and valuable avenue for exploration. Notably, unlike agricultural crop residues, the utilization of *S. salsa* presents a unique advantage by eliminating resource competition concerns with the livestock industry. While agricultural crop residues play a pivotal role as feed in the livestock sector, the utilization of *S. salsa* offers an innovative alternative for pigment extraction and other applications, providing a distinctive solution without resource conflicts.

This study explored the co-production of beet red pigments and BNC from halophyte *S. salsa*. Pigment extraction and characterization were carried out, and the chemical and physical effects of the extraction process on the *S. salsa* biomass were explored. The enzymatic digestibility of the *S. salsa* biomass before and after the extraction was compared. The resulting hydrolysates were also compared regarding their BNC fermentability. BNC produced from *S. salsa* hydrolysates was further evaluated through scanning electron microscopy observation, ATR-FTIR spectroscopy analysis, X-ray diffraction techniques and thermal stability analysis. The efficient co-production process and the beneficial effects of the pigment extraction procedure on cellulase saccharification and BNC fermentation were investigated.

## 2 Materials and methods

### 2.1 Materials

*S. salsa* biomass was harvested from Binzhou Port in Shandong Province. Diatomaceous earth was procured from the Shaanxi Institute of Mineralogical and Chemical Research. Sorghum vinegar was purchased from Kelan County, Xinzhou City, Shanxi Province. Glucose, peptone, yeast extract, sodium hydroxide, and concentrated sulfuric acid were purchased from Sinopharm Chemical Reagent Co., Ltd. Cellulase was purchased from Shanghai Yuanye Bio-Technology Co., Ltd., A microporous adsorption resin was purchased from Beijing Solarbio Science and Technology Co., Ltd.

### 2.2 Pigment extraction and characterization

The aboveground parts of *S. salsa* were washed, milled, and pretreated with 1% (w/v) NaOH aqueous solution at 50°C for 2 h before the pigment extraction. The addition of 1% (w/v) NaOH effectively facilitated the release of pigments, thereby improving the efficiency of pigment extraction. The pretreated material was extracted twice using ddH_2_O with solid fraction of 5% (w/w) at 50°C for 90 min. The filtrate was collected and centrifuged at 5,000 revolutions per minute (r/min) for 15 min. The supernatant was then retrieved, and clarifying diatomaceous earth was added at a concentration of 2 g/L. After shaking the mixture evenly, it was allowed to settle for 15 min. Following this, a second centrifugation at 5,000 r/min for an additional 15 min was conducted. The solid residual was collected by filtration and dried at 60°C for 12 h. Beet red pigment from *S. salsa* was separated and purified of with macroporous resin. The pigment was diluted with ddH_2_O, and full wavelength scanning was conducted within the wavelength ranges of 190–650 nm and 340–650 nm to determine the maximum absorption wavelength using a ultraviolet spectrophotometer (NewCentury, Beijing Purkinje GENERAL Instrument Co., Ltd.).

### 2.3 Chemical analysis of composition of *S. salsa* before and after the pigment extraction

The *S. salsa* materials were milled prior to the analysis. The lignin and carbohydrate contents of the *S. salsa* samples were analyzed according to the ASTM E1758-01 method (Yagmur et al., 2020). ICP-MS (NexION 300, PerkinElmer, United States) was used to determine the contents of mineral and heavy metal elements. The measurements were all performed in triplicate.

### 2.4 Pretreatment

Appropriate amounts of NES and ES were dried in a constant temperature oven at 110°C for 6 h. The dried materials were weighed and added to a 2% sulfuric acid solution, with a solid-to-liquid ratio of 1:20 (Wang et al., 2018). These samples were then placed in triangular flasks and subjected to pretreatment at 115°C for 25 min in a high-pressure sterilizer. After the pretreatment, the pH of the samples was adjusted to 5.0 using NaOH at room temperature (25°C).

### 2.5 Enzymatic saccharification

The cellulase (S10041, YUANYE) were sterilized using 0.22 μm filters before used, and the pretreated samples were hydrolyzed with cellulase with final enzyme activity of 125 U/mL and 250 U/mL. The samples were then placed in a temperature-controlled shaking incubator (TS-2102, JTLIANGYOU) at 45°C and 140 rpm for 48 h (Ilanidis et al., 2021). After the enzymatic saccharification, the supernatant was collected by centrifugation at 8,000 r/min for 5 min, and the glucose content and pH of the solution were measured.

### 2.6 Culture medium

The fermentation media for LDU-A and LDU-K are glucose-based (glucose-based culture medium), consisting of 20.0 g/L glucose, 5.0 g/L peptone, and 3.0 g/L yeast extract (Chen et al., 2018). The strains LDU-A and LDU-K used in the study were named based on the abbreviation “LDU” representing our work place “Ludong University,” and A or K were the initials of the locations from which the samples originated. The fermentation medium for LDU-A and LDU-K strains were: the control DYPD medium, 3.5 g/L glucose, 5.0 g/L peptone, and 3.0 g/L yeast extract; non-extracted *S. salsa* medium (NES-M), hydrolysate from the non-extracted *S. salsa* was adjusted to a final glucose concentration of 3.5 g/L (based on the glucose released from the enzymatic saccharification experiments), peptone 5.0 g/L, yeast extract 3.0 g/L; extracted *S. salsa* medium (ES-M), hydrolysate from the extracted *S. salsa* was adjusted to a final glucose concentration of 3.5 g/L, peptone 5.0 g/L, yeast extract 3.0 g/L. The final pH was all adjusted to 5.0.

### 2.7 BNC strain isolation and characterization

Strain LDU-A was previously isolated from rotten apples from Aksu, Xinjiang in our laboratory (unpublished results) and identified with 16s rRNA sequence as *Komagataeibacter intermedius* accession number KT894755. A strain named LDU-K was isolated from sorghum vinegar produced in Kelan County, Xinzhou City, Shanxi Province. The gelatinous cellulose floating on the surface of sorghum vinegar was collected and washed with distilled water in a sterile environment. It was then transferred to the glucose-based culture medium (20 g/L glucose, 5 g/L peptone, 3 g/L yeast powder), and cultured at 30°C for 5 days. When gelatinous cellulose was formed, 100 μL culture was taken and was diluted for 10^4^, 10^5^, and 10^6^ times with ddH_2_O, and was spread on glucose-based agar culture medium (agar: 20 g/L). After cultured at 30°C for 7 days, the white culture colonies surrounded by white transparent circles was picked and placed in DYPD liquid medium for verification. The 16S ribosomal RNA (rRNA) was sequenced, and a BLAST search was conducted using the NCBI nucleotide database. The generated data were compared to the closest matches, and the Clustal W multiple aligners were utilized to identify consensus regions. Molecular phylogenetic analysis was conducted using MEGA software (version 6) for systematic evolutionary analysis. A neighbor-joining method was employed to construct the phylogenetic tree, with numerical values representing bootstrap values (%).

### 2.8 Bacteria salt tolerance test

The salt sensitivity of the bacteria was tested using test medium, in which 0%, 0.5%, 0.75%, 1.0%, and 1.5% NaCl were added to the glucose-based culture medium (Ho Jin et al., 2019), respectively. LDU-A and LDU-K were incubated statically at 30°C for 10 days. Samples were taken at the beginning of the fermentation and for every 2 days for glucose consumption evaluations. The produced BNC was collected and purified at the end of the fermentation. The experiments were repeated independently for three times.

### 2.9 BNC fermentation

All bacterial strains were pre-cultured in the glucose-based culture medium under static conditions at 30°C for 5 days. During this process, bacteria exist in a non-metabolically active state. Through a pre-cultivation period of 5 days, the bacteria can be revived, initiating a reactivation of growth. The strain was then inoculated into the glucose-based culture medium (4.0%, v/v) and incubated statically at 30°C for 10 days (Chen et al., 2018). The produced BNC membranes were rinsed with water to remove residual culture medium and then soaked in deionized water. The water was changed every 1 hour for 5 times to obtain the crude-washed BNC. The crude-washed BNC was then washed with 0.1 mol/L NaOH solution at 80°C for 4 hours. Finally, the washed BNC membranes were rinsed with deionized water at 80°C until the pH reached neutral. After the purification process, the BNC was dried at 60°C until a constant weight was achieved, and then weighed.

### 2.10 Analysis on glucose

Analysis of glucose was performed by using high-performance liquid chromatography (HPLC) and a glucometer. A Bio-rad Aminex HPX-87H Column (7.8 × 300 mm) was used in an Agilent 1,260 Infinity series system (Agilent, Santa Clara, CA, United States) equipped with a 1,260 series diode array and multiple wavelength (DAD) detector and a 1,260 series refractive index (RI) detector. Elution was performed with isocratic flow of a 0.005 M aqueous solution of sulfuric acid. The flow rate was 0.6 mL/min and the column temperature was set to 55°C. Agilent software was used for data analysis. Glucometer was also used for initial measurements of glucose. Calibration curves was made, and the glucose content was calculated based on the calibration curve.

### 2.11 Characterization

#### 2.11.1 Scanning electron microscopy analysis

The *S. salsa* materials before and after the pigment extraction were analyzed using a field emission scanning electron microscope (FE-SEM) (Sigma500, ZEISS). The materials were fixed and gold-plated before imaging. Images magnified at 1,000 and 20,000 times were taken. The purified and dried BNC samples were analyzed using the same microscope and method, and images magnified at 20,000 times were taken.

#### 2.11.2 Fourier infrared spectroscopy analysis

The BNC samples were subjected to Perkin Elmer FTIR spectrophotometer (Thermo Nicolet 6,700, NEXUS, TM) equipped with an attenuated total reflection (ATR) assembly with a zinc selenide (ZnSe) crystal. 16 scans of each sample within a range from 4,000 to 500 cm^−1^ at a 4 cm^−1^ resolution were collected.

#### 2.11.3 X-ray diffractometer analysis

The BNC raw material was analyzed using a Rigaku Smart LabSEx X-ray diffractometer (XRD) from Japan, employing CuKα1 radiation (λ = 0.154178 nm). XRD data was collected in the 2θ range of 5°–90°, with a step size of 0.02° and a scanning rate of 10°/min. The Jade software was utilized for phase identification and data analysis, referencing the diffraction patterns provided by the International Center for Diffraction Data (ICDD).

#### 2.11.4 Thermogravimetric analysis

Thermogravimetric analysis (TGA) was conducted using a METTLER TGA2 weight analyzer. BNC samples were first weighed and placed in a tray with an initial weight of 3–5 mg. The samples were heated from 25°C to 800°C at a constant heating rate of 10°C/min under N_2_ environment.

## 3 Results and discussion

### 3.1 Pigment extraction

**FIGURE 1:**
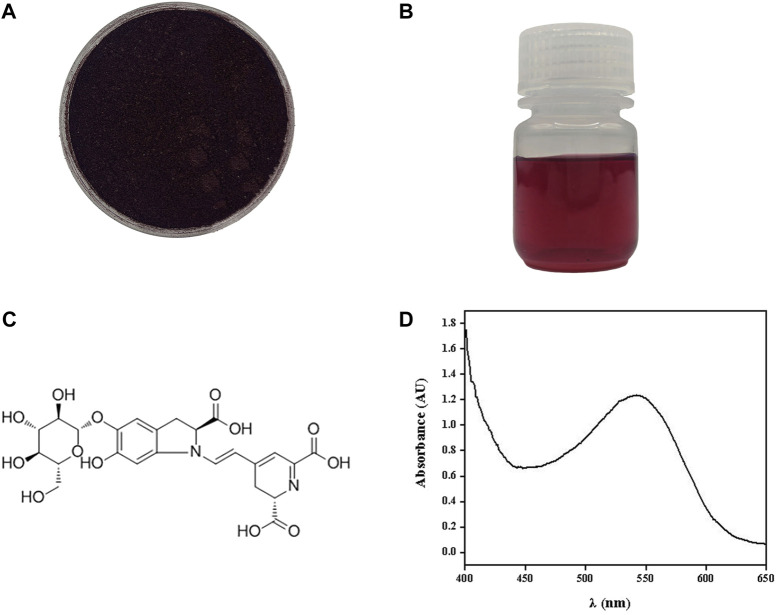
Beet red pigment extracted from *S. salsa*. Photograph of beet red pigment powder **(A)** and beet red pigment H_2_O solution **(B)**. Molecular formula of the main component betaine **(C)**. Absorption spectrum of beet red pigment **(D)**.

The beet red pigment was successfully extracted from *S. salsa*. After rotary evaporation, a soluble powder with a distinct dark red-colored appearance was obtained (Figure 1A). It dissolved in water at a concentration of 0.67 g/L resulting in a solution displaying a crimson color (Figure 1B). A full-wavelength scan of the extracted material was conducted, revealing a prominent absorption peak for beet red pigment at 542 nm (Figure 1D). Chauhan reported that the primary component of beet red pigment was betanin (Figure 1C), constituting 75%–95% of the betacyanin. It existed in two forms, free and glycosylated, while the remaining components included isobetanin, prebetanin, isoprebetanin, and degradation products of betacyanin (Chauhan et al., 2013).

The lignocellulosic composition, salts and heavy metal contents, and total flavonoid content were measured (Table 1). The sodium concentration in our study closely mirrors the findings of LI et al., demonstrating a high degree of similarity (Li et al., 2017). After the pigment extraction, only 1.8% of the total beet red pigment was left in the residue. At the same time, the content of Na^+^, K^+^, Mg^2+^, Fe^2+^, Fe^3+^, Cr^2+^, Cr^3+^, Cr^6+^, and Cl^−^ were reduced by at least 89.1%. And the total flavonoids content of the samples also decreased by 82.7% after the extraction. On the contrary, the ES sample exhibited higher cellulose (39.97%) and hemicellulose (39.74%) contents, which were not soluble and were enriched during the extraction. The pre-treatment (extraction) process of *S. salsa* plants had enhanced enzymatic hydrolysis efficiency, The reduced salt and total flavonoid contents were advantageous for enzymatic saccharification and fermentation.

**TABLE 1:** The content of beet red pigment, cellulose, hemicellulose, lignin, mineral and total flavonoids (w%).

*S. Salsa* material	Beet red pigment	Cellulose	Hemicellulose	Lignin	Na^+^	K^+^	Mg^2+^	Fe (Fe^2+^, Fe^3+^)	Cr (Cr^2+^,Cr^3+^ Cr^6+^)	Cl^−^	Total flavonoids
NES	2.82± 0.22	33.14 ± 0.82	34.23 ± 1.63	11.35 ± 0.32	5.11 ± 0.22	0.96 ± 0.07	0.42 ± 0.02	0.09 ± 0.01	0.03 ± 0.01	4.35 ± 0.26	2.26 ± 0.19

ES	0.05± 0.01**	39.97 ± 1.27	39.74 ± 0.66	13.21 ± 0.39	0.13 ± 0.02**	0.03 ± 0.01**	0.02 ± 0.01**	0.01 ± 0.01	ND^a^**	0.08 ± 0.01**	0.39 ± 0.02**


**p < 0.01.

**FIGURE 2:**
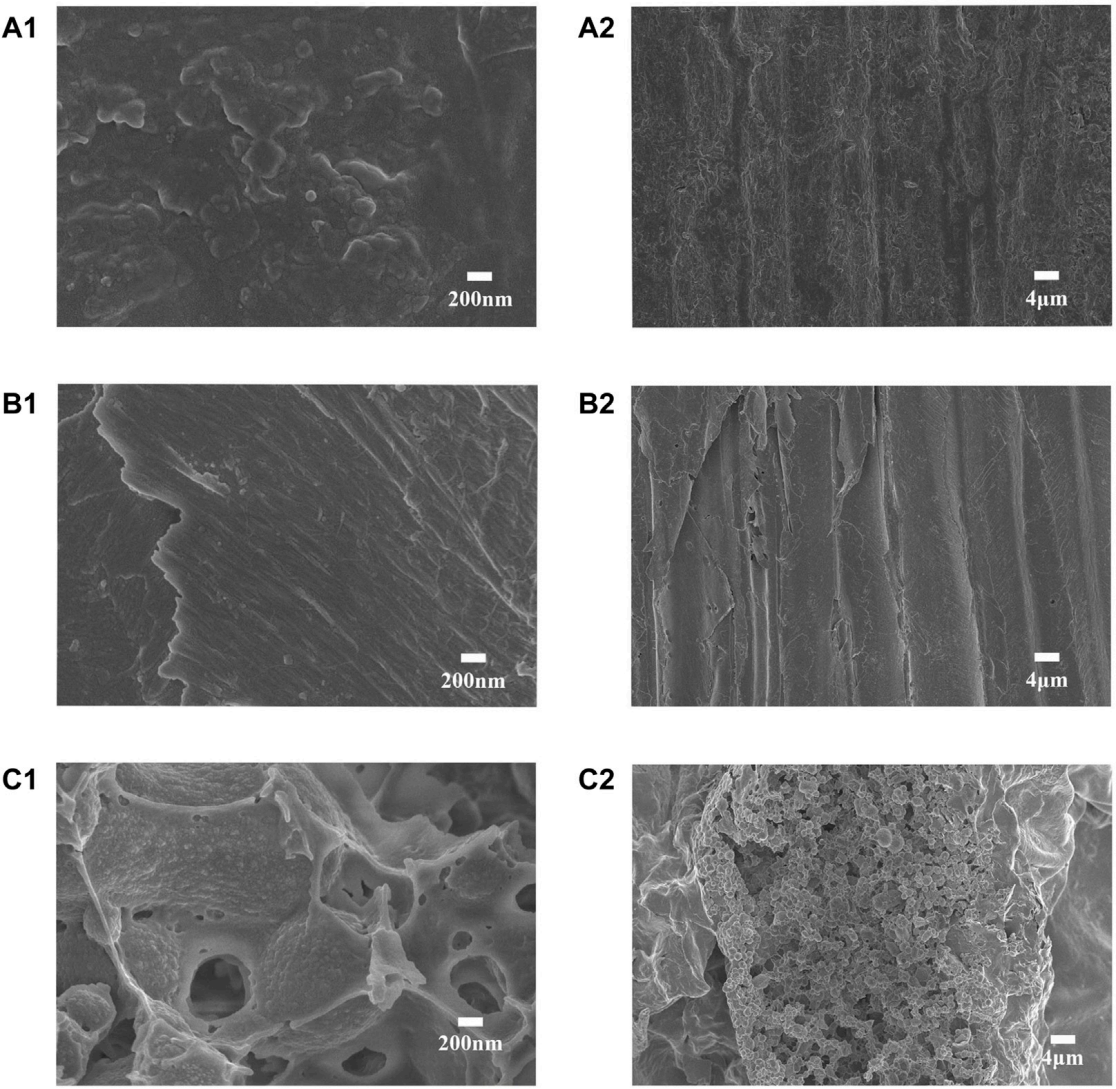
The scanning electron microscopy (SEM) images of the *S. salsa* materials before and after the pigment extraction: The outer epidermis of *S. salsa* straw, magnified by ×20000 **(A1)** and ×1,000 **(A2)**, respectively; *S. salsa* straw interior part magnification ×20,000 **(B1)** and ×1,000 **(B2)**; the *S. salsa* residue after the pigment extraction, magnified ×20,000 **(C1)** and ×1,000 **(C2)**.

In this study, we scanned the outer epidermis and inner diameter of the *S. salsa* plant and the residue after the pigment extraction for the microstructure of the samples (Figure 2). The outer epidermis of *S. salsa* plant presented a rough, split, and textured morphology (A1, A2), while the inner part presented a compact and arranged fiber structure (B1, B2), which contributed to the strength and rigidity of the straw. The residue of the *S. salsa* plant after the pigment extraction became loose and porous (C1, C2), due to the physical and chemical treatments during the process of the pigment extraction. The loose and porous structure made the residue more accessible to cellulase (Deng et al., 2016), resulting in much more efficient enzymatic saccharification than that of the *S. salsa* biomass before the pigment extraction (Table 2).

**TABLE 2:** Glucose released from the enzymatic saccharification of the *S. salsa* materials before and after the pigment extraction.

*S. Salsa* material	Pretreatment condition	Cellulase Ⅰ^a^ (U/mL)		Cellulase Ⅱ^b^ (U/mL)	
		24 h	48 h	24 h	48 h
NES	H_2_O	ND^c^	ND	ND	ND
	H_2_SO_4_	0.40 ± 0.04	0.94 ± 0.07	0.44 ± 0.05	1.01 ± 0.09
ES	H_2_O	ND	ND	ND	ND
	H_2_SO_4_	4.17 ± 0.16	4.59 ± 0.12	4.36 ± 0.24	4.79 ± 0.49

^a^ Cellulase Ⅰ, 125 U/mL cellulase.

^b^ Cellulase Ⅱ, 250 U/mL cellulase.

^c^ ND, none Detected.

### 3.2 Enzymatic saccharification

The results of the enzymatic saccharification of the *S. salsa* materials before and after the pigment extraction was shown in Table 2, the glucose released from ES was 10.4 and 4.9 times higher than that from NES after 24 h and 48 h, respectively. During the pigment extraction process, the compact structure of the straw was partially damage, making it more accessible to cellulase (Figure 2). Moreover, during the multiple washing steps during the pigment extraction, significant portion of non-cellulosic substances were removed, including iron and phenolic compounds (Table 2).

Lignocellulosic biorefineries heavily relied on effective pretreatment strategies to enhance the conversion efficiency of enzymatic hydrolysis. In this context, Li et al. employed a combination of biological pretreatment with deep eutectic solvent pretreatment to enhance the enzymatic saccharification of Pinus massoniana (Li et al., 2023). Additionally, McIntosh et al. achieved enhanced enzyme saccharification of Sorghum bicolor straw by utilizing a dilute alkali pretreatment approach, Alkaline pretreatment successfully delignifies biomass by disrupting the ester bonds cross-linking lignin and xylan, resulting in cellulose and hemicellulose enriched fractions (McIntosh and Vancov, 2010). In our study, utilized a rare alkali pretreatment and washing method for pigment extraction. This process not only disrupted the ester linkages between lignin and hemicellulose, successfully achieving delignification, but also effectively removed a substantial amount of inhibitory substances through the washing steps, including iron ions and flavonoid compounds. This approach significantly improved the efficiency of saccharification and BNC fermentation. The rationale behind this improvement lies in the fact that high concentrations of certain ions and compounds have the potential to compete with enzyme substrates or cofactors, thereby inhibiting cellulase activity, as elucidated by Zhai et al., in 2015 (Zhai et al., 2015). Finally, the glucose released from NES and ES using 250 U/mL cellulase was only improved for about 7.4% and 4.4%, respectively, than using 125 U/mL cellulase. As a result, using 125 U/mL cellulase was more economical. And the hydrolysates with 125 U/mL cellulase was use in the following BNC fermentation experiments.

### 3.3 BNC strain isolation and identification

**FIGURE 3:**
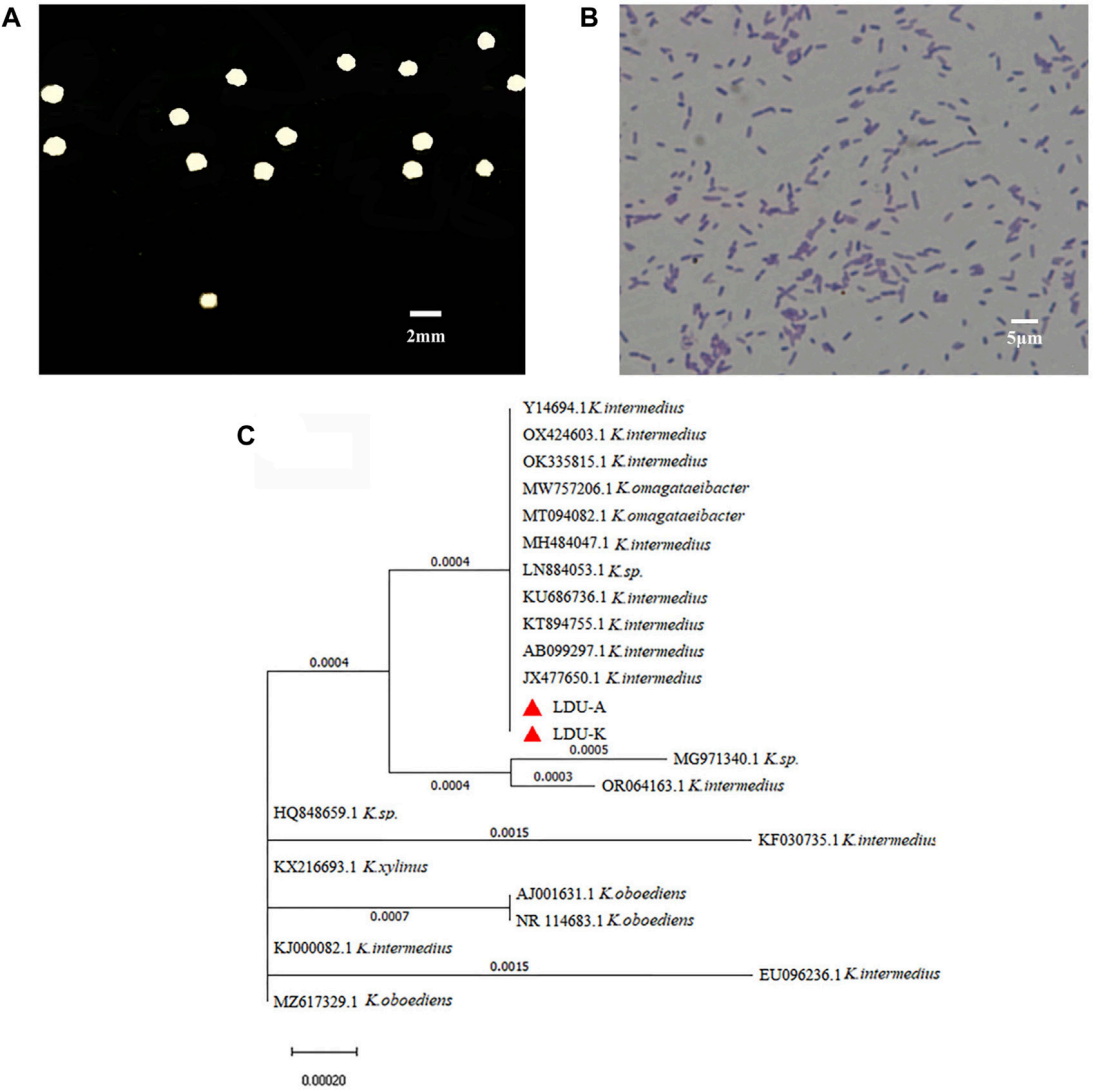
Characteristics of LDU-K strain. Colony of strain LDU-K on agar plate **(A)** and image of microscope after gram staining **(B)** Phylogenetic tree **(C)** based on 16S rRNA sequence shows the relationship of strains LDU-A and LDU-K to their immediumte neighbors.

In the current study, a new BNC producing strain was isolated from sorghum vinegar, and was name as LDU-K. The LDU-K colony had a white circular bulge surrounded by a white transparent circle (Figure 3A), and the bacterium was Gram-negative (Figure 3B). Based on sequence analysis of the 16S rRNA gene, it was revealed by NCBI that the highest similarities to members of the family *Komagataeibacter* and the strain was closely related to *K.intermedius* accession number JX477650 (100% similarity) (Figure 3C). *K. intermedius* isolates were isolated from various sources, including fruit juice (Lin et al., 2016), wine vinegar (Fernández et al., 2019), pear peel, pomace (Ma et al., 2021) and kombucha tea (Dos Santos et al., 2015), and were widely used in BNC fermentation (Cannazza et al., 2022; Devanthi et al., 2022). In the current study, LDU-K, as well as another *K. intermedius* strain LDU-A, which was isolated previously in our laboratory (unpublished results), was used to evaluate the fermentability of the *S. salsa* hydrolysates.

### 3.4 Bacteria salt sensitive test

**FIGURE 4:**
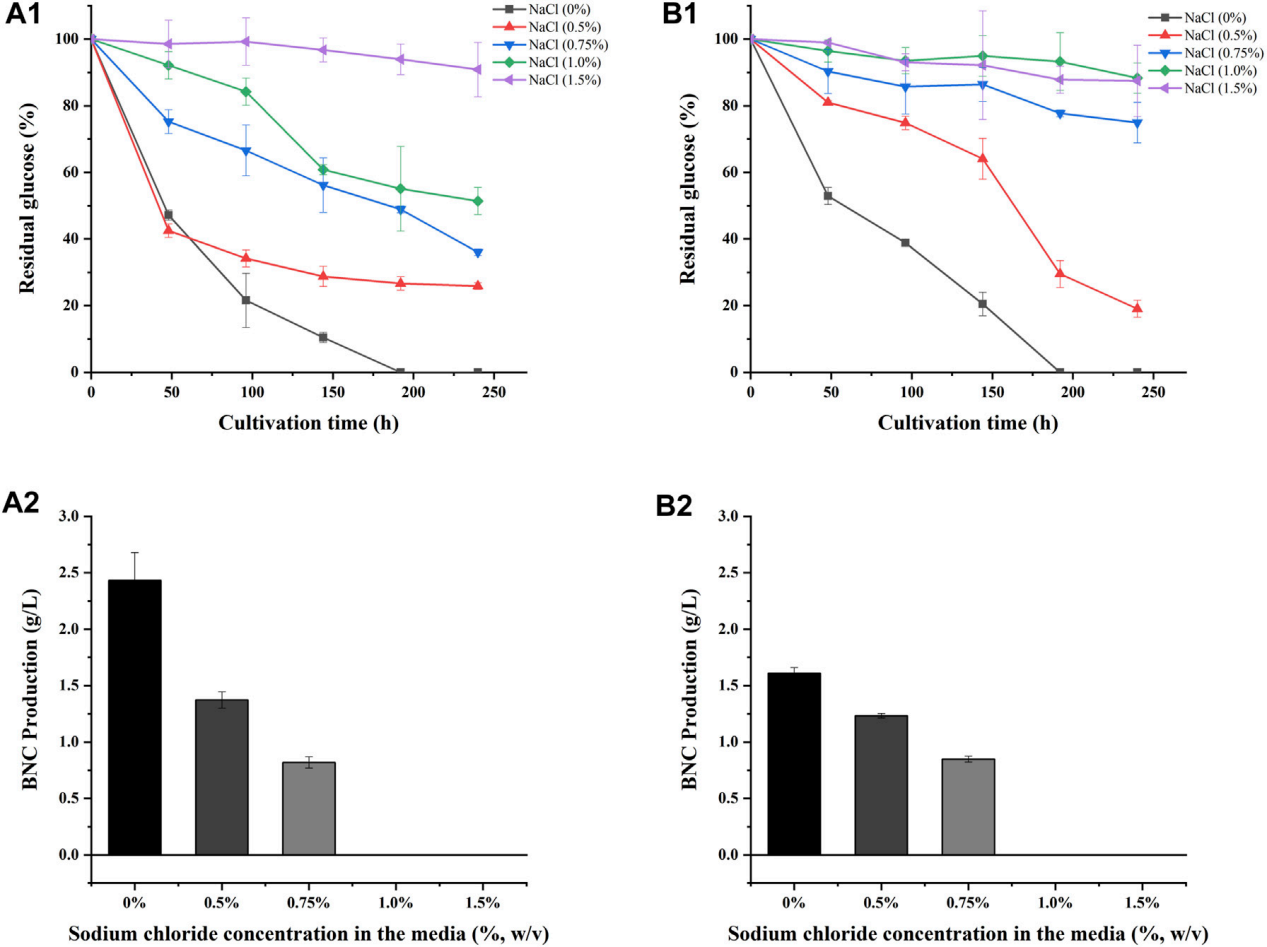
Sodium chloride tolerance of LDU-A and LDU-K. Glucose consumption of LDU-A **(A1)** and LDU-K **(B1)** in culture medium with sodium chloride concentration gradients; BNC production of LDU-A **(A2)** and LDU-K **(B2)** in culture medium with sodium chloride concentration gradients.

As *S. salsa* straw contained large amounts of salts and was used as a carbon source for BNC fermentation in this study, experiments on the salt tolerance of the bacteria LDU-A and LDU-K were conducted. The glucose consumption and BNC yield were shown in Figure 4. Within the first 72 h, bacteria in the culture medium without NaCl proliferated significantly, leading to a rapid glucose consumption, and a small amount of BNC membrane was produced. However, in the culture medium containing 0.75%, 1%, and 1.5% salt, glucose consumption was significantly slower or non-existent. By the eighth day of cultivation, glucose was completely depleted in the 0% salt-containing medium, while there was still residual glucose in all the salt-containing medium. As the salt concentration increased, more glucose remained unconsumed (Figure 4). As regard to the BNC production of LDU-A and LDU-K, when the salt content in the culture medium was 0.5%, the yield decreased by 88.2% and 83.3%, respectively. At 0.75% salt content, the yield decreased by 93.4% and 94.9%. When the salt content reached 1%, BNC production ceased entirely. The highest productivities were observed in culture medium without NaCl (Table 3), and were 0.014 g/(L × h) and 0.012 g/(L × h) for LDU-A and LDU-K, respectively. For both strains, BNC productivity, glucose consumption, and glucose conversion rate all decreased as the salt content in the culture medium increased. The BNC production can be greatly influenced by bacterial species, type of reactor, pH, temperature, aerobic conditions, and concentration of the nutrients in the culture medium (Dórame-Miranda et al., 2019). The results indicated that the bacteria were both hyper-sensitive to the saline environments, and NaCl greatly inhibited the BNC production (Ho Jin et al., 2019). As the saline plant *S. Salsa* biomass contained substantial amounts of salts (Shi et al., 2020), the reduction of salt content before BNC fermentation is essential.

**TABLE 3:** Glucose consumption rates, BNC productivity, and BNC (P, product) yields of initial and consumed glucose (Glc, glucose) of LDU-A and LDU-K in presence of NaCl^a^.

Culture medium with NaCl content gradients		Productivity [g/(L × h)]	Glucose consumption rate [g/(L × h)]	^b^Y_P/initial Glc_ (g/g)	^c^Y_P/consumed Glc_ (g/g)
LDU-A	0%	0.014 ± 0.001	0.083 ± 0.009	0.162 ± 0.003	0.162 ± 0.003
	0.5%	0.002 ± 0.001	0.062 ± 0.009	0.019 ± 0.005	0.026 ± 0.006
	0.75%	0.001 ± 0.001	0.053 ± 0.018	0.011 ± 0.003	0.017 ± 0.004
	1.0%	ND^d^	0.041 ± 0.012	ND	ND
	1.5%	ND	0.008 ± 0.002	ND	ND
LDU-K	0%	0.012 ± 0.001	0.083 ± 0.001	0.143 ± 0.002	0.143 ± 0.002
	0.5%	0.002 ± 0.001	0.067 ± 0.013	0.024 ± 0.002	0.020 ± 0.003
	0.75%	0.001 ± 0.001	0.021 ± 0.003	0.007 ± 0.002	0.030 ± 0.006
	1.0%	ND	0.010 ± 0.001	ND	ND
	1.5%	ND	0.010 ± 0.008	ND	ND

^a^ Results based on 10-day old cultures.

^b^ Y_P/initial Glc_, BNC, conversion rate of the initial glucose.

^c^ Y_P/consumed Glc_, BNC, conversion rate of the glucose consumed.

^d^ ND, none detected.

### 3.5 BNC fermentation

**FIGURE 5:**
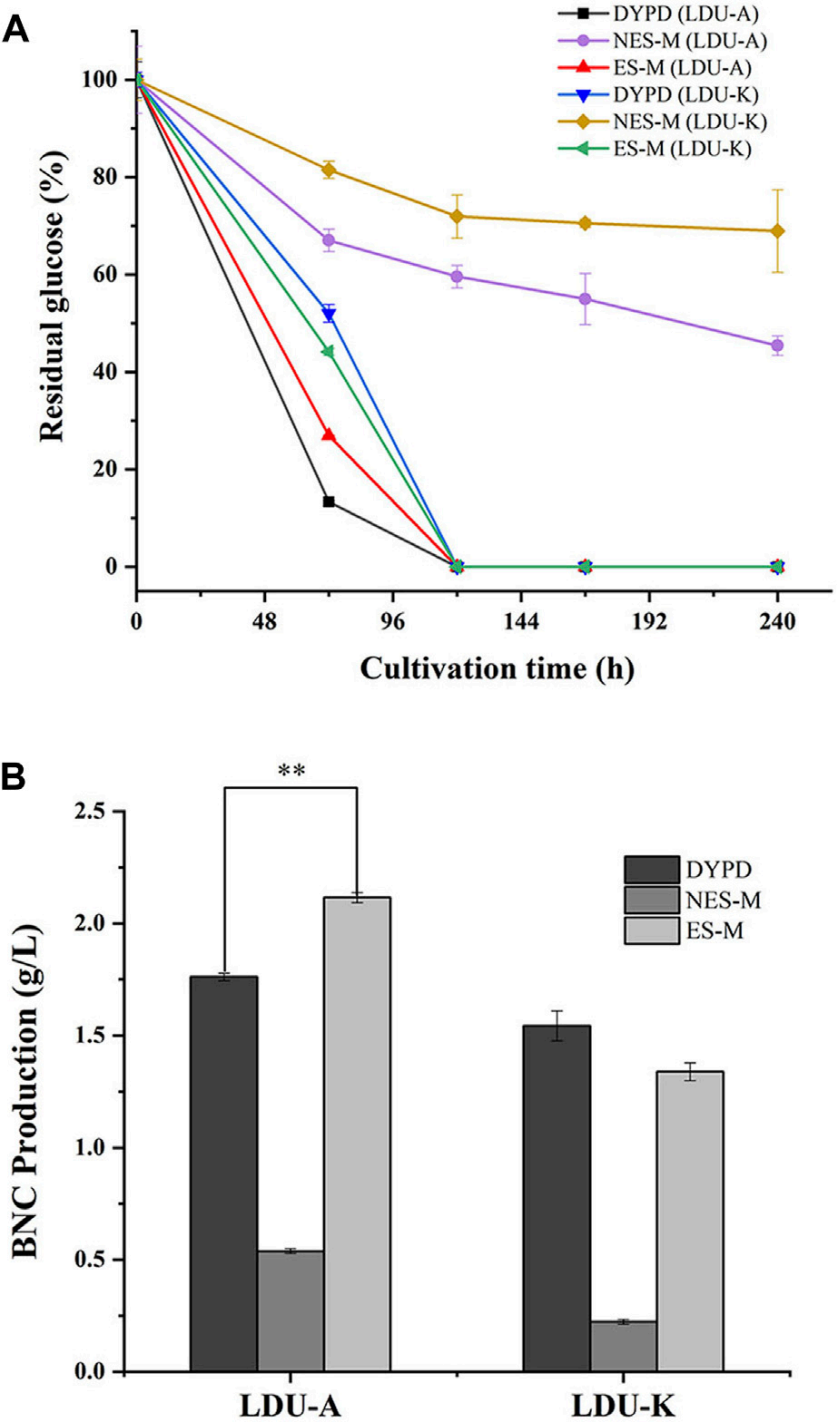
**(A)** Glucose consumption of LDU-A and LUD-K in DYPD, NES-M and ES-M. **(B)** BNC yield of LDU-A and LDU-K in DYPD, NES-M, and ES-M.

Glucose serves as both the energy source for bacteria and a prerequisite for cellulose production during BNC synthesis (Castro et al., 2011). In this study, DYPD medium and two types of *S. salsa*-based medium (NES-M and ES-M) were used. The glucose consumption rates of LDU-A were higher than that of LDU-K in all the three mediums. Within the first 3 days, LDU-A and LDU-K consumed 73.1% and 55.9% of glucose in DYPD, respectively. LDU-A consumed 86.7% of glucose, while LDU-K consumed 48.0% in ES-M. In NES-M, LDU-A and LDU-K consumed 40% and 28% of glucose, respectively. By day 5 of the cultivations, glucose was completely consumed in DYPD and ES-M, while large amounts of glucose remained in NES-M (Figure 5A). BNC production was measured after 10 days, and the results showed that LDU-A produced 20.0% more BNC from ES-M than that from DYPD and the yield was 2.116 g/L, while LDU-K achieved 86.7% of the control group’s BNC yield and the yield was 1.339 g/L. However, in NES-M, the BNC yield was lower, and LDU-A and LDU-K produced only 30.6% and 14.5% of those from DYPD, the yields were 0.539 g/L and 0.223 g/L, respectively (Figure 5B). The results also showed that LDU-A had the highest productivity and glucose conversion rate in ES-M (Table 4). The BNC productivity of LDU-A and LDU-K in NES-M was only 22.2% and 16.7% of that in ES-M, indicating that pigment extraction greatly improved the fermentability of the *S. salsa* hydrolysates.

**TABLE 4:** Glucose consumption rate, BNC productivity, and BNC (P, product) yields of initial and consumed glucose (Glc, glucose) in static cultivations^a^.

Fermentaion strain and medium		Yield (g/L)	Productivity [g/(L × h)]	Glucose consumption rate [g/(L × h)]	^b^Y_P/initial Glc_ (g/g)	^c^Y_P/consumed Glc_ (g/g)
LDU-A	DYPD	1.762 ± 0.017	0.007 ± 0.001	0.020 ± 0.001	0.374 ± 0.003	0.374 ± 0.004
	NES	0.539 ± 0.001	0.002 ± 0.001	0.011 ± 0.001	0.114 ± 0.001	0.210 ± 0.001
	ES	2.116 ± 0.023	0.009 ± 0.001	0.020 ± 0.001	0.450 ± 0.004	0.450 ± 0.005
LDU-K	DYPD	1.543 ± 0.067	0.006 ± 0.001	0.018 ± 0.001	0.354 ± 0.015	0.354 ± 0.015
	NES	0.223 ± 0.001	0.001 ± 0.001	0.005 ± 0.001	0.051 ± 0.001	0.174 ± 0.001
	ES	1.339 ± 0.040	0.006 ± 0.001	0.018 ± 0.001	0.307 ± 0.009	0.307 ± 0.001

^a^ Results based on 10-day old cultures.

^b^ Y_P/initial Glc_, BNC, conversion rate of the initial glucose.

^c^ Y_P/consumed Glc_, BNC, conversion rate of the glucose consumed.

Glucose was an important component of cellulose structure, and the BNC producing strains utilized glucose for cell growth and metabolic activities (Cocinero et al., 2009; Li et al., 2015). In ES-M, the bacteria were able to fully utilize glucose, whereas complete utilization of glucose was challenging in NES-M. As both LDU-A and LDU-K were hyper-sensitive to NaCl, we speculated that the presence of ions such as Na^+^, K^+^, Mg^2+^, Cl^−^, and SO_4_^2−^ in NES-M from *S. salsa* might affect glucose utilization and BNC fermentation. Studies have been conducted on production of BNC utilizing biomass or waste materials as carbon sources. Huang et al. employed wastewater after lipid fermentation as raw material to synthesize BNC, achieving a yield of 0.4 g/L (Huang et al., 2016). Dorame-Miranda et al. explored the use of walnut shells as substrate, yielding 0.82 g/L of BNC (Dorame-Miranda et al., 2019). Lin et al. isolated *K. intermedius* from fermented fruit juice for BNC production, achieving an impressive yield of 1.2 g/L (Lin et al., 2016). Cakar et al. investigated the feasibility of utilizing molasses for BNC production, achieving an impressive yield of 1.64 g/L (Cakar et al., 2014). Martínez et al. conducted research utilizing fig juice as a carbon source, resulting in a noteworthy yield of 2.2 g/L (Martínez et al., 2023). In our investigation, similar or more BNC was produced using the *S. salsa* biomass, and at the same time, beet red pigment was also produced. The *S. salsa* residue after pigment extraction could be used as an exceptional and cost-effective carbon source, and our study offered an eco-friendly and economically co-production process for pigment production and BNC synthesis.

### 3.6 BNC characterization

**FIGURE 6:**
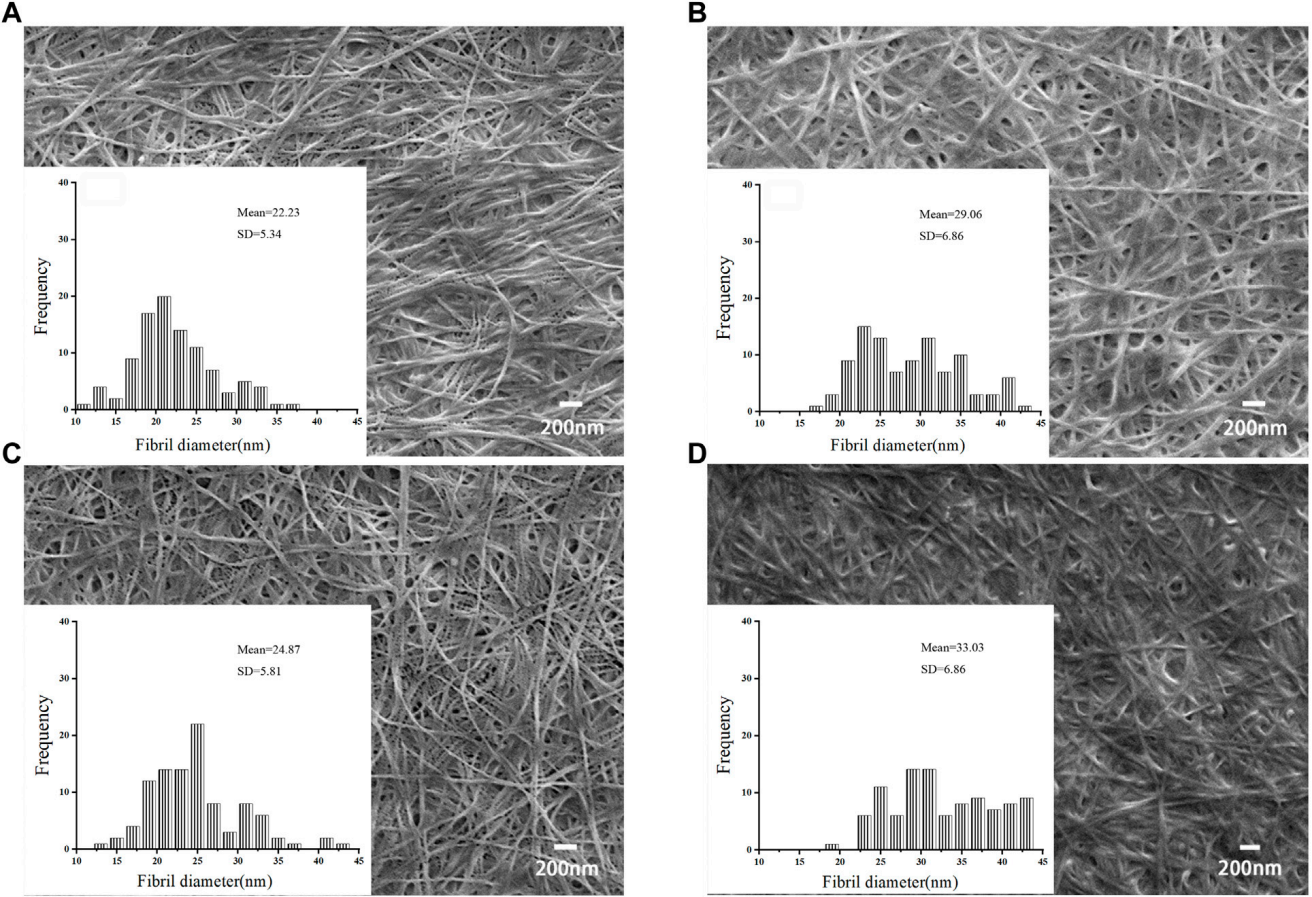
SEM images and fiber diameters of BNC produced from DYPD and ES-M. The SEM images of BNC produced by LDU-A from DYPD (A1) and ES-M (B1), and BNC produced by LDU-K from DYPD (C1) and ES-M (D1). Zoom in ×500,00.

The morphology of BNC during static cultivation were determined using scanning electron microscopy (SEM). The average fiber diameters of the four BNC materials in this study were from 22.23 to 33.03 nm (Figure 6), falling within the similar range as reported by other researchers, displaying the typical structure of nanocellulose fibers. The diameters of BNC fiber produced in ES-M were 29.06–33.03 nm, which was larger than that produced in DYPD (22.23–24.87 nm). The carbon source of ES-M was more complex. In addition to glucose, ES-M contained xylose and mannose, etc., which might affect the synthesis of BNC. Zhang reported an average BNC fiber diameter of 15.7 nm using atomic force microscopy (Zhang, 2013). Algar reported nanofibers with a width of 30–40 nm prepared using pineapple waste (Algar et al., 2015). Ruka reported that under dynamic cultivation conditions, BNC microfibrils had diameters ranging from 24 to 55 nm (Ruka et al., 2012). Similarly, Khan and Abol-Fotouh reported BNC nanofiber diameters ranging from 10 to 90 nm when supplementing HS medium with fig extract and date extract, respectively (Abol-Fotouh et al., 2020; Khan et al., 2021). Comparison with other studies highlights distinctions in our results. Specifically, our findings differ from those of K. Saleh et al. (Saleh et al., 2022), who obtained BC membrane nanofibers with diameters ranging from 62.14 nm to 83.94 nm under different cultivation conditions. Additionally, El-Gendi et al. reported an average nanofiber diameter of 41.10 nm for BC membranes cultivated in HS medium (El-Gendi et al., 2023), while PPPE-generated PPPE-BC membranes exhibited an average fiber diameter of 91.64 nm. These variations may be attributed to differences in bacterial strains and medium composition, a normal occurrence in bacterial cellulose research. The average diameters of the BNC produced using ES-M by both LDU-A and LDU-K were in the ranges of the BNC fiber diameters in previous reports.

**FIGURE 7:**
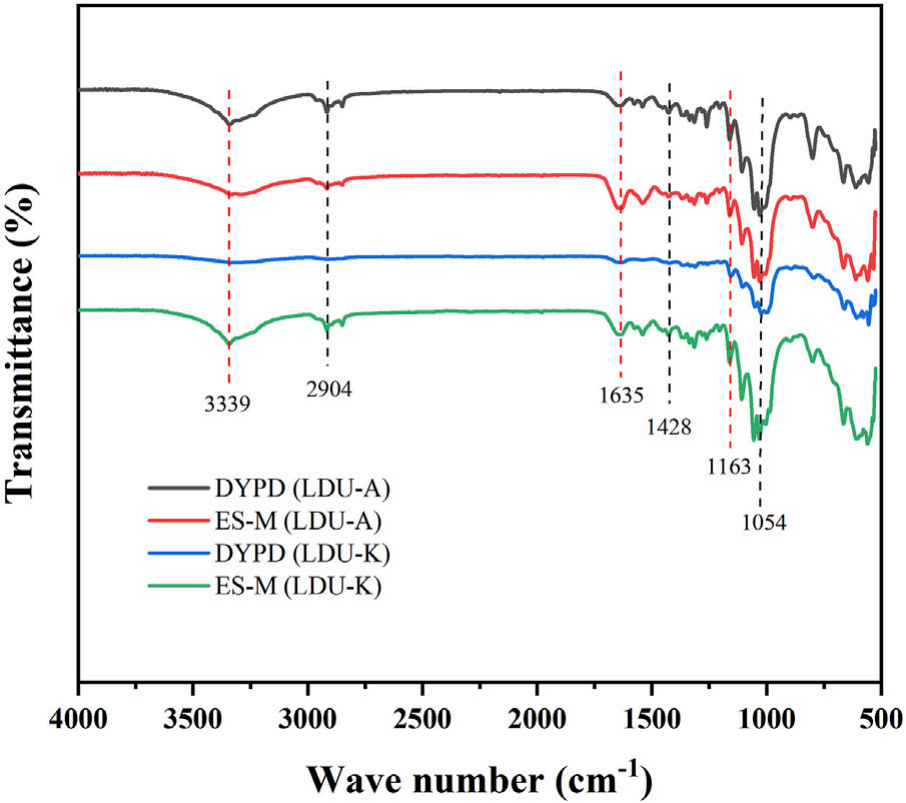
FTIR spectra of BNC produced by LDU-A and LDU-K using DYPD and ES-M.

The four BNC samples produced by LDU-A and LDU-K all exhibited similar spectral features and characteristic peaks related to cellulose subjected to Fourier-transform infrared spectroscopy (FTIR) analysis. BNC is primarily an unbranched macromolecular linear polymer composed of pyranose glucose monomers linked by β-1,4-glycosidic bonds (Chen et al., 2019). It exhibits an abundance of functional groups, including -OH and C-H. The peaks observed at wave numbers 3339 cm^−1^ and 2,904 cm^−1^ correspond to the tensile vibrations of -OH and C-H, respectively. The wave number at 1,635 cm^−1^ is attributed to the bending vibration of the hemiacetal group atop the glucose molecule (Figure 7). The bending vibration of CH2 manifests at 1,428 cm^−1^. Additionally, telescopic motion of C-O is evident at wave numbers 1163 cm^−1^ and 1,054 cm^−1^. These results were consistent with the characterization of the samples as cellulose, and there were no significant differences among the four BNC preparations. Our results agreed with previous studies regarding the chemical groups in several BNC samples and highlights the distinctive features of cellulose (Oh et al., 2005; Yassine et al., 2016).

**FIGURE 8:**
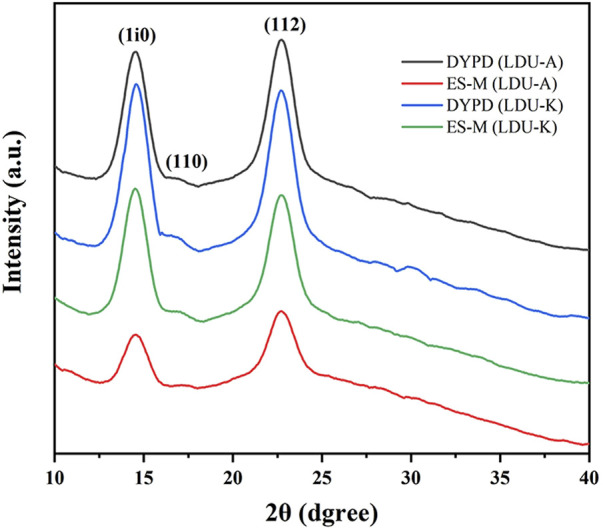
XRD patterns of BNC produced by LDU-A and LDU-K using DYPD and ES-M.

The XRD analysis was conducted. The results were presented in Figure 8, which depicted three distinct peaks observed at 2θ = 14.5°, 16.7°, and 22.7°. These peaks corresponded to the diffractions of the (101), (10i), and (002) crystallographic planes of the cellulose structure, as determined using the XRD peak-fitting method. Further analysis was undertaken to determine the interplanar spacing (d-spacing), size of each crystalline plane (ACS), and peak broadening (FWHM). The analysis ultimately facilitated the calculation of the crystallinity index (CrI) and the identification of the primary cellulose phase (Iα or Iβ) (Thorat and Dastager, 2018). The results were shown in Table 5, and the crystallinity of the BNC material in this study ranged from 77% to 90%. The crystallinity of BNC produced by LDU-K in DYPD was the highest (82%), and the crystallinity of BNC produced by LDU-A in ES-M was the lowest (77.30%). Overall, the crystallinity of BNC prepared in ES-M was slightly lower than that produced in DYPD. We speculated that the lower crystallinity was related to the higher yield of BNC (Figure 5B). Due to the faster BNC synthesis of LDU-A in ES-M, and the fiber synthesis might be carried out before the formation of the denser fiber crystals. Through comprehensive analysis, our BNC samples were identified to possess the Iα crystalline phase. Our results agreed with previous findings that the composition of the culture medium influenced the crystallinity of the produced BNC (Castro et al., 2012; Khan et al., 2021).

**TABLE 5:** Crystallinity of BNC produced by LDU-A and LDU-K using DYPD and ES-M.

Fermentaion strain and medium		d-spacing (nm)			ACS (nm)			FWHM (2θ)			CrI (%)	DA
		(1i0)	(110)	(112)	(1i0)	(110)	(112)	(1i0)	(110)	(112)		
LDU-A	DYPD	0.610	0.532	0.391	5.63	5.19	3.57	1.49	1.49	1.67	89.00	Iα
	ES-M	0.608	0.520	0.391	6.43	4.45	4.15	1.30	1.70	1.73	77.30	Iα
LDU-K	DYPD	0.608	0.531	0.391	5.51	6.73	3.70	1.52	1.25	1.64	89.72	Iα
	ES-M	0.610	0.528	0.391	5.50	4.81	3.50	1.48	1.74	1.69	88.04	Iα

**FIGURE 9:**
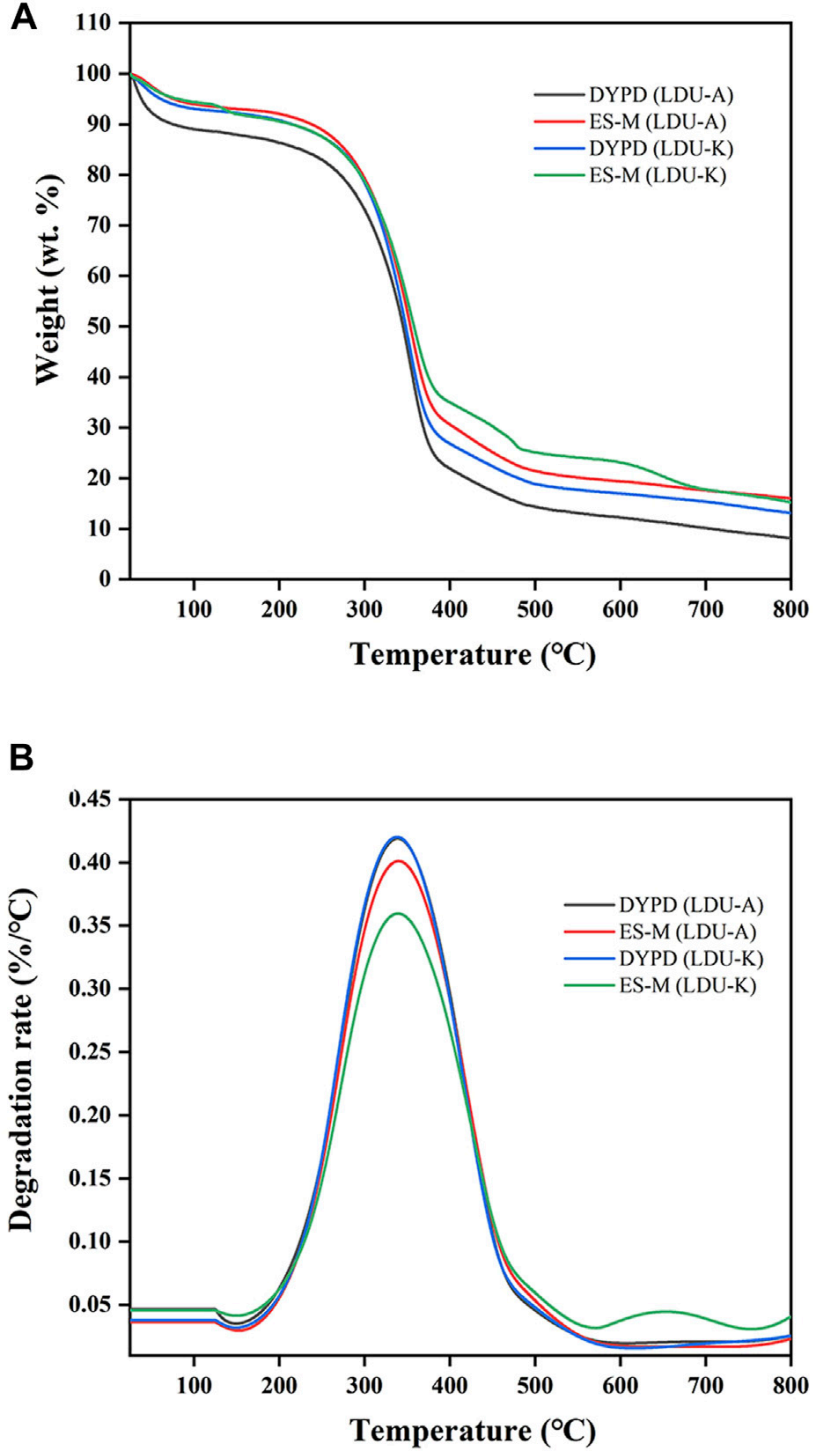
Thermogravimetric **(A)** and differential thermogravimetric **(B)** curves of BNC produced by LDU-A and LDU-K from DYPD and ES-M.

The results of TGA analysis and differential thermogravimetric (DTG) analysis were shown in Figure 9; Table 6. The TGA results revealed three distinct stages of continuous weight loss. The first stage was with a sharp decline occurred at 30°C–100°C, corresponded to the initial stage of moisture evaporation, and reached relatively stable states with nearly constant weights. The second stage with a rapid decline was observed at 240°C–380°C, which is slightly higher than the temperature range of 300°C–350°C reported by Castro et al. (Castro et al., 2012) but similar to 300°C–370°C reported by Dubey et al. (Dubey et al., 2018). The rapid weight loss in this stage was attributed to the degradation of cellulose, including depolymerization, dehydration, and decomposition of glucose units, where the samples experienced weight loss ranging from 50% to 59%. The temperature range of the final stage was 400°C–800°C corresponded to the formation of carbonaceous residues resulting from the oxidation of char. In this experiment, the TGA curves of the BNC produced in ES-M and NES-M exhibited slight differences compared to the BNC produced in glucose-based medium. The variations might be attributed to the presence of sugars other than glucose in ES-M and NES-M. In the temperature range of 224°C–458°C, the four BNC materials exhibited degradation rates exceeding 0.1%/°C, and degradation rates exceeding 0.25%/°C occurred between 269°C and 412°C. BNC produced by the two strains using glucose as the carbon source and ES displayed the highest weight loss rate at 339°C. In comparison, other researchers reported the maximum weight loss rate of economically sourced BNC at 310°C, and BC produced using HS medium exhibited the maximum decomposition at 290°C (Costa et al., 2017). Overall, the thermal degradation temperature range of all BNC materials in this study remains stable, exhibiting consistent thermal degradation trends, and demonstrating higher thermal stability compared to other researchers, all the four samples exhibited similar degradation trends.

**TABLE 6:** The thermal degradation performance of BNC produced by LDU-A and LDU-K using DYPD and ES-M.

Fermentaion strain and medium		DTG peak (°C)	Degradation rate at DTG peak (%/°C)	^a^Degradation rate >0.1(%/°C)	^a^Degradation rate >0.25 (%/°C)
LDU-A	DYPD	338	0.42	224–452	270–412
	ES-M	339	0.41	227–451	269–410
LDU-K	DYPD	338	0.42	227–456	273–411
	ES-M	339	0.36	229–458	280–406

^a^ Degradation rate, the temperature range when BNC, weight loss rate >0.1%/°C or >0.25%/°C.

## 4 Conclusion

This study developed a novel approach of co-production of pigment and BNC from *S. salsa* biomass. The extraction washed away the main parts of the salts and flavonoids, promoting both the cellulase saccharification for 10.4 times and the BNC fermentation up to 4 times. SEM revealed the disrupted lignocellulosic fiber structure, and the chemical analysis revealed the decreased cellulase and BNC fermentation inhibitors. BNC produced from *S. salsa* was characterized, and was found to have smaller crystal size. This novel discovery not only facilitates the comprehensive utilization of *S. salsa* but also addresses the challenges of high costs and low enzymatic conversion efficiency in biomass pretreatment and enzymatic saccharification processes. Simultaneously, it enables the combined production of pigments and BNC, significantly enhancing economic output. Our study shed light on the biorefinery of saline-alkali plants. The salt resistant bacteria for high BNC production warrants further investigation.

## Data Availability

The original contributions presented in the study are included in the article/Supplementary material, further inquiries can be directed to the corresponding authors.
